# Efficacy of Immunostimulatory Adjuvants and Nano‐Adjuvants in Current SARS‐CoV‐2 Vaccines: A Comprehensive Review

**DOI:** 10.1002/hsr2.71405

**Published:** 2025-11-17

**Authors:** Peyman Kheirandish Zarandi, Mohammad Reza Zinatizadeh, Mohsen Ghiasi, Mohammad Rezaei Zadeh Rukerd, Hanieh Mirkamali, Ehsan Shokri

**Affiliations:** ^1^ Department of Biology, Science and Research Branch Islamic Azad University Tehran Iran; ^2^ Cancer Biology Signaling Pathway Interest Group (CBSPIG) Universal Scientific Education and Research Network (USERN) Tehran Iran; ^3^ Cardiovascular Research Center Rajaie Cardiovascular Institute Tehran Iran; ^4^ Department of Stem Cells and Developmental Biology, Cell Science Research Center Royan Institute for Stem Cell Biology and Technology, ACECR Tehran Iran; ^5^ Faculty of Medicine Kerman University of Medical Sciences Kerman Iran; ^6^ Department of Nanotechnology, Agricultural Biotechnology Research Institute of Iran (ABRII), Agricultural Research Education and Extension Organization (AREEO) Karaj Iran

**Keywords:** immunization, nano‐adjuvant, nanotechnology, SARS‐CoV‐2, vaccination

## Abstract

**Background and Aims:**

SARS‐CoV‐2 continues to pose global challenges, and current vaccines face limitations (variant escape, waning immunity). This review evaluates immunostimulatory adjuvants and nano‐adjuvants to enhance immune responses and optimize SARS‐CoV‐2 vaccine performance.

**Methods:**

Narrative review of preclinical and clinical evidence on alum, emulsions (MF59, AS01/AS03), Montanide ISA‐51, delta inulin, TLR agonists (e.g., CpG, TLR3), rOv‐ASP‐1, and nano/mucosal platforms (liposomes, polymeric or metal nanoparticles, Protollin) for SARS‐CoV‐2 and related coronaviruses.

**Results:**

Alum and emulsions increased neutralizing antibodies; Th1‐promoting combinations (e.g., CpG with Montanide or with protein antigens) mitigated Th2‐biased immunopathology seen with some inactivated vaccines. MF59, AS01/AS03, and Matrix‐type systems enhanced humoral and cellular responses, with early clinical data supporting acceptable safety and robust immunogenicity. Delta inulin (±CpG) boosted neutralization without lung injury in comparative models. TLR agonists and intranasal Protollin induced systemic IgG and mucosal IgA. Nano‐adjuvants improved antigen presentation and enabled dose‐sparing while supporting balanced, durable immunity.

**Conclusion:**

Immunostimulatory and nano‐adjuvants substantially strengthen SARS‐CoV‐2 vaccine immunogenicity, support antigen‐sparing, and favor balanced (often Th1‐biased) protection. Prudent adjuvant selection and integration of conventional and nanotechnology‐derived platforms are key to achieving safe, durable, and broadly protective COVID‐19 vaccines.

**Abbreviations**
ACE2: angiotensin‐converting enzyme 2
Ad26.COV2.S: adenovirus type 26‐based COVID‐19 vaccine
APC: antigen‐presenting cell
ARDS: acute respiratory distress syndrome
AuNP: gold nanoparticle
CD8⁺ T cells: cluster of differentiation 8 positive T cells
ChAdOx1: chimpanzee adenovirus oxford 1
CoV: coronaviruse
COVID‐19: coronavirus disease 2019
CpG 1018: CpG oligodeoxynucleotide 1018
CpG ODN: CpG oligodeoxynucleotide
DI (Advax): delta inulin
E protein: envelope protein
HA: hemagglutinin
hDPP4: human dipeptidyl peptidase 4
HIV‐1: human immunodeficiency virus type 1
HLA‐A*0201*
: *human leukocyte antigen A*0201
IFN‐β: interferon beta
IFN‐γ: interferon gamma
IgA: immunoglobulin A
IgG: immunoglobulin G
IgG1: immunoglobulin G subclass 1
JEV: Japanese encephalitis virus
LNP: lipid nanoparticle
LPS: lipopolysaccharide
MERS‐CoV: Middle East respiratory syndrome coronavirus
MHC: major histocompatibility complex
mRNA: messenger RNA
M protein: membrane protein
NVX‐CoV2373: Novavax COVID‐19 vaccine candidate
N protein: nucleocapsid protein
PAMP: pathogen‐associated molecular pattern
PIKA: polyinosinic‐polycytidylic acid
PRR: pattern recognition receptor
QS‐21: *Quillaja Saponaria* Saponin 21
RBD: receptor‐binding domain
ROS: reactive oxygen species
rOv‐ASP‐1: Recombinant Onchocerca volvulus Activation‐Associated Protein‐1
S1: spike protein subunit 1
S2: spike protein subunit 2
SARS‐CoV: severe acute respiratory syndrome coronavirus
SARS‐CoV‐2: severe acute respiratory syndrome coronavirus 2
S protein: spike protein
TDCM: trehalose dicorynomycolate
Th2: T helper type 2 cells
TLR: toll‐like receptor
TMPRSS2: transmembrane serine protease 2
UV: ultraviolet
VLP: virus‐like particle
+ssRNA: positive‐sense single‐stranded RNA
β‐CoV: beta coronavirus

## 1 Introduction

Severe acute respiratory syndrome coronavirus (SARS‐CoV)−2, a member of the family Coronaviridae and the causal agent of coronavirus disease 2019 (COVID‐19) illness, has rapidly spread throughout the world since the first case in Wuhan, China, in December 2019 [1, 2, 3]. Despite the extensive measures implemented to contain the virus, its spread continues. Even though most cases cure spontaneously, some patients experience catastrophic complications, such as severe pneumonia, organ failure, and acute respiratory distress syndrome (ARDS) [4, 5, 6, 7, 8].

Two related viruses responsible for similar diseases, SARS‐CoV and Middle East respiratory syndrome coronavirus (MERS‐CoV), emerged in 2003 and 2012, respectively, causing significant damage. The SARS‐CoV‐2 is recognized as a β‐coronavirus (β‐CoV) and, like the SARS‐CoV agent, attaches to angiotensin‐converting enzyme 2 (ACE2) receptors [9, 10]. At the nucleotide level, more than 70% similarity was observed between the SARS‐CoV‐2 and SARS‐CoV genomes [10, 11]. SARS‐CoV‐2, a positive‐sense single‐stranded RNA (+ssRNA) virus, encodes the spike (S), envelope (E), membrane (M), and nucleocapsid (N) proteins [12]. There is a 72% nucleotide similarity between the two viruses in the spike protein that attaches to host cell receptors [13, 14, 15, 16]. The S protein comprises S1 and S2, with S1 interacting with the cell surface receptor and S2 mediating fusion of viral and cell membranes to facilitate virus entry into host cells. Various CoVs use the S1 receptor‐binding domain (RBD) to bind to host cell receptors [10, 13]. The RBD of MERS‐CoV recognizes nonacetylated sialoside receptors, whereas SARS‐CoV interacts with ACE2 receptors [17, 18, 19]. The SARS‐CoV‐2 RBD contains different amino acids compared with SARS‐CoV, which are essential for high‐affinity binding [20]. In addition, SARS‐CoV‐2 has a functional polybasic furin cleavage site at the S1‐S2 junction, which may play a role in determining tissue tropism and enhancing infection [21, 22]. In SARS‐CoV, a furin cleavage site is absent, and fusion with the host cell membrane occurs via receptor‐mediated direct fusion or receptor‐mediated endocytosis [23]. The rapid outbreak and high capability for person‐to‐person transmission of this virus can be attributed to the strong binding affinity of SARS‐CoV‐2 to the ACE2 receptor and the presence of furin and transmembrane serine protease 2 (TMPRSS2). Although specific antiviral drugs for SARS‐CoV‐2 were not available during the initial phase of the pandemic, various therapeutic options have since been developed and authorized. These include antivirals, such as remdesivir, molnupiravir, and nirmatrelvir/ritonavir (Paxlovid), which have been authorized for clinical use to reduce viral replication and improve patient outcomes [24, 25]. Additionally, innovative drug delivery systems like ACEI‐capped remdesivir‐loaded PLGA nanoparticles designed through computational simulations offer promising approaches to enhance anti‐SARS‐CoV‐2 therapy [3]. These therapeutic advances collectively represent important strategies against COVID‐19 and its complications.

**Figure 1:**
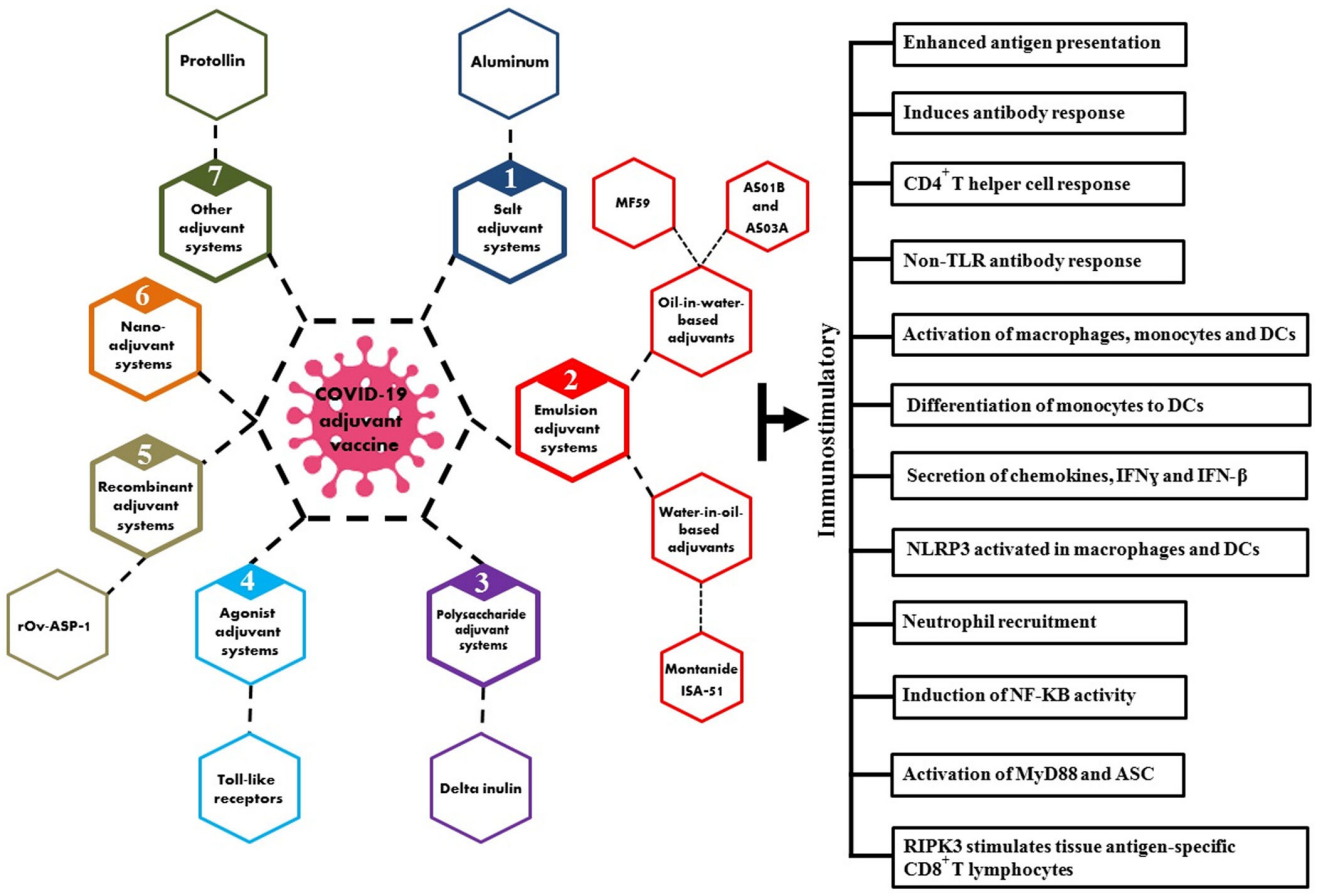
A schematic representation illustrates the innate and adaptive immune responses activated by various adjuvants.

Due to the similarities among SARS‐CoV‐2, SARS, and MERS coronaviruses, compiling therapeutic information about these viral agents will be useful in developing the best COVID‐19 treatment process. Vaccine candidates against SARS‐CoV that have undergone extensive testing include inactivated whole‐virus vaccines, recombinant S protein formulations, virus‐like particles (VLPs), plasmid DNA vaccines, and several viral vectors encoding SARS‐CoV proteins [26, 27, 28]. Most available vaccines have elicited neutralizing antibodies [29]. These antibodies are designed to target the coronavirus spike protein and prevent the virus from entering cells [30, 31]. It is well established that higher antibody titers correlate with improved therapeutic efficacy of vaccines, underscoring the importance of strategies that enhance these responses. However, several limitations remain in developing optimal vaccines, including reduced effectiveness against emerging SARS‐CoV‐2 variants, waning immunity requiring booster doses, partial prevention of viral transmission, and challenges in achieving herd immunity due to uneven vaccine coverage and heterogeneous immune responses across populations. Concerns about long‐term safety and operational difficulties in global vaccine distribution also persist [32]. When developing the optimal vaccine, efficacy should be prioritized, and safety should be maximized. Therefore, the use of immunostimulatory adjuvants in vaccine development can mitigate these current limitations by enhancing and prolonging immune protection, improving vaccine efficacy, and optimizing safety profiles [30, 31]. The innate and adaptive immune responses elicited by various adjuvants are illustrated in Figure 1.

The optimal vaccine should be able to induce protective antibody responses with a minimal antigen dose as quickly as possible and without causing harm. Although widespread vaccination efforts have mitigated much of the immediate COVID‐19 burden, the ongoing emergence of variants and the inherent possibility of future pandemics caused by novel infectious agents underscore the critical need for vaccines that confer rapid, broad, and durable protection with minimal antigen usage. Maintaining preparedness for the rapid development and deployment of such vaccines is essential to effectively counter emerging threats and meet both current and future global needs [33, 34]. Identifying the optimal adjuvants for the COVID‐19 vaccine will enhance resistance to the disease. Furthermore, developing the optimal vaccine will reduce antigen usage and boost immune system activation. The aim of this review is to assess immunostimulatory and nano‐adjuvants in SARS‐CoV‐2 vaccines, highlighting promising strategies to enhance immune responses and optimize vaccine performance.

## 2 Salt Adjuvant Systems

### 2.1 Aluminum

Aluminum hydroxide (alum) is widely used in immunostimulation. It is not yet clear how it works, but studies suggest that alum causes a slow diffusion of antigen by forming a depot at the injection site. The function of this agent prolongs the interaction time between antigen and antigen‐presenting cells (APCs) and enhances phagocytosis of antigens [35]. At the cellular level, aluminum hydroxide directly stimulates monocytes, inducing pro‐inflammatory cytokine release and activating T cells. T helper type 2 (Th2) cells secrete interleukin (IL)‐4 and increase the expression of major histocompatibility complex (MHC) class II molecules on monocytes [36]. T helper type 1 (Th1) and Th2 responses are two key branches of adaptive immunity with distinct roles. Th1 responses promote cell‐mediated immunity, activating macrophages and cytotoxic T cells to fight intracellular pathogens, mainly through cytokines like interferon‐gamma (IFN‐γ). Th2 responses, in contrast, support humoral immunity by aiding B cells in antibody production, especially through cytokines such as IL‐4. Maintaining a balanced Th1/Th2 response is critical for effective and safe immune protection; an excessive Th2 bias can lead to adverse effects like antibody‐dependent enhancement (ADE), while a strong Th1 response generally ensures durable immunity and reduces immunopathology [37]. This balance is especially important in vaccine development to optimize efficacy and safety against SARS‐CoV‐2.

Alum adjuvant is approved for use in several vaccines [38, 39]. Notably, Sinopharm, a Chinese pharmaceutical company, uses aluminum salts as an adjuvant in its inactivated COVID‐19 vaccine [40]. Long‐term production of memory B cells and sustained antibody secretion have been observed in SARS‐CoV vaccine experiments. Experiments have shown that including alum in the vaccine increases immunoglobulin G (IgG) levels in immunized mice [41]. In the experiments, the antibodies recognized spike and nucleocapsid proteins and were able to neutralize the virus. Further induction of SARS‐CoV antibodies by adjuvanted vaccines has been demonstrated [42]. In nonhuman mammals, both attenuated SARS‐CoV vaccines with and without alum demonstrated adequate protection. Moreover, in MERS‐CoV attenuated vaccines, the combination of alum and CpG also prevented the disease [43, 44, 45]. Although the safe and effective use of inactivated viruses in vaccines has been recognized, some experiments have shown that antibody responses and immunopathological changes in the lungs after injection can lead to pathogenesis [46, 47, 48]. Additional studies have suggested that nonneutralizing antibodies, antibody isotype, and differences in the affinity or levels of inactivating antibodies in inactivated virus vaccines may lead to these immunopathological variations [48, 49, 50, 51].

Adjuvants that induce Th1 reactions in vaccines containing inactivated viruses can reduce Th2‐type immunopathological variations [45, 48, 52]. These findings can inform the development of an optimal vaccine. In combination with alum, a truncated form of the SARS‐CoV glycoprotein S elicits much higher levels of neutralizing antibodies at lower doses, and a lower amount of glycoprotein S has been shown [53, 54]. Various forms of deglycosylated RBD proteins were expressed as novel combinations in yeast. The inclusion of alum in experiments significantly enhanced RBD immunoreactivity and induced a high level of neutralizing antibodies against SARS‐CoV [55]. New combination preparations of spike protein and alum were as effective as VLPs containing the spike protein and the influenza M1 protein; both vaccine formulations performed well in mouse experiments by inducing a strong antibody response [56]. VLPs comprising the SARS‐CoV S protein and the E, M, and N proteins of murine hepatitis virus have been used in experiments on Balb/c mice [57]. The incorporation of alum in VLP formulations increased the production of neutralizing antibodies and protected mice against SARS‐CoV. In experiments on mice, formulations comprising MERS‐CoV, SARS‐CoV antigens, and stimulant nanoparticles were able to induce high levels of antibodies [58]. Similarly, MERS‐CoV alum nanoparticles and a new combination of MERS‐CoV RBD vaccines can induce neutralizing antibodies and defend Rhesus macaques [59, 60].

## 3 Emulsion Adjuvant Systems

### 3.1 Oil‐in‐Water‐Based Adjuvants

#### 3.1.1 MF59

MF59 is an oil‐in‐water adjuvant composed of squalene and two surfactants, Tween 80 and Span 85. It is used in influenza pandemic vaccines [61, 62]. It has been employed in many antibacterial and antiviral vaccines, including the SARS‐CoV vaccine. Higher antibody levels are induced when this adjuvant is combined with the vaccine [63, 64, 65]. Like alum, it potentiates Th2 responses, but it can also activate more CD4+ T cells and enhance antibody production against the HA influenza virus [66]. In the vaccine tested on mice, this adjuvant demonstrated the ability to protect against SARS‐CoV and induced neutralizing antibodies after two doses [67]. S1 protein‐based vaccines and MERS‐CoV RBD epitopes combined with MF59 significantly increased IgG titers and neutralizing responses. This combination protected mice against the virus [68, 69, 70]. These experimental vaccines have induced neutralizing antibodies and protected human dipeptidyl peptidase 4 (hDPP4)‐transgenic mice against the disease [70, 71]. The hDPP4 transgenic mice represent an essential small animal model for the study of coronaviruses, particularly MERS‐CoV. Unlike conventional small animals such as mice, hamsters, and ferrets, which lack the hDPP4 receptor necessary for viral entry, hDPP4 transgenic mice express this receptor, rendering them susceptible to infection. This susceptibility enables productive viral replication and the development of clinical manifestations, including acute pneumonia that closely mimics human disease. The use of hDPP4 transgenic mice circumvents the limitations associated with nonhuman primate models, including high costs, limited availability, complex husbandry requirements, and ethical constraints. Infection through aerosol exposure in these models accurately replicates natural respiratory transmission pathways, offering a solid foundation for the study of viral pathogenesis, immune responses, and the preclinical assessment of antiviral therapies and vaccine candidates. Collectively, hDPP4 transgenic mice constitute an invaluable tool in advancing coronavirus research and facilitating the development of effective medical countermeasures [72].

Importantly, human phase 1 clinical trial data for an MF59‐adjuvanted recombinant SARS‐CoV‐2 spike glycoprotein‐clamp vaccine confirm its safety and strong immunogenicity. In a randomized, double‐blind, placebo‐controlled study involving healthy adults aged 18–55, the vaccine was well tolerated with predominantly mild local and systemic adverse events and no serious safety concerns. This vaccine elicited robust antigen‐specific IgG and neutralizing antibody titers exceeding those observed in convalescent COVID‐19 patients, alongside strong CD4^+^ T cell responses. Moreover, sera from vaccinated individuals neutralized SARS‐CoV‐2 variants of concern, underscoring the vaccine's potential efficacy [73].

#### 3.1.2 AS01B and AS03A

The oil‐in‐water adjuvant systems AS01B and AS03A are manufactured by GlaxoSmithKline (GSK). The AS01 adjuvant contains liposome‐based monophosphoryl lipid A (MPL) and a saponin molecule (QS‐21). The MPL and QS‐21 are derived from Salmonella Minnesota and the bark of the South American *Quillaja Saponaria* Molina, respectively. MPL signaling via the Toll‐like receptor 4 (TLR4) activates APCs and produces cytokines and interferons (IFNs). On the other hand, QS‐21 is known to enhance antigen‐specific antibody production, which pertains to humoral immunity, as well as to promote cellular immunity through activation of APCs and T‐cell responses. Therefore, QS‐21 contributes to both humoral and cellular arms of the adaptive immune system, leading to robust and balanced vaccine‐induced immunity [74, 75]. When combined with the new SARS‐CoV S protein, QS‐21 elicits high serum antibody titers and protection against infection [76]. Recently, AS01 has been used in the malaria vaccine RTS,S/AS01 and tested in herpes zoster, the human immunodeficiency virus (HIV) type 1 polyprotein vaccine, and the tuberculosis vaccine [77, 78, 79, 80]. This system was also used to evaluate a vaccine based on the attenuated SARS‐CoV virus in mice. In these experiments, it was found that AS01B elicited a slightly stronger immune response than AS03A, and overall, the immune effects of these vaccines in animals were enhanced. It is noteworthy that after vaccination, the SARS‐CoV agent did not cause additional destructive effects on the lungs or liver of hamsters [81].

### 3.2 Water‐in‐Oil‐Based Adjuvants

#### 3.2.1 Montanide ISA‐51

Montanide, a water‐in‐oil emulsion, consists of mineral oil and a surfactant from the mannide monooleate group. This agent stimulates the immune system by forming a depot at the injection site, which leads to a slower release of antigens, local inflammation, and enhanced recruitment of APCs. It increases antibody titers and enhances the activity of cytotoxic T lymphocytes against the associated antigen [82, 83].

This agent has been tested with varying degrees of success in vaccines for acquired immunodeficiency syndrome (AIDS) and malaria, and it is better tolerated in humans compared with Freund's incomplete adjuvant (FIA) [84, 85, 86, 87]. FIA primarily induces immune responses characterized by Th2‐type cytokine production, which promotes antibody‐mediated immunity. It functions by forming a depot at the injection site, enabling slow antigen release and prolonged stimulation of APCs and other innate immune cells. Although IFA mainly drives humoral immunity, it can elicit a mixed cytokine environment with variable Th1 and Th2 responses depending on the context [88]. The combination of Montanide ISA‐51 and CpG ODN has been used in vaccine formulations to enhance Th1 immune responses. Montanide ISA‐51 and CpG are used to emulsify a novel formulation of the N protein of the SARS‐CoV virus. In experiments, strong Th1 responses have been observed in mice and macaques following this formulation [89]. The combination of synthetic peptides of SARS‐CoV and Montanide ISA‐51/CpG with spike proteins has been used to produce potent antibodies that, in animal experiments, have prevented the entry of SARS‐CoV pseudovirus into HepG2 cells [90]. The DNA vaccine encoding the SARS‐CoV N protein, in combination with Montanide ISA‐51/CpG, has been shown to elicit a robust anti‐N immune response [91]. Adequate titers of virus‐neutralizing antibodies have been observed in experiments combining the novel RBD of MERS‐CoV with Montanide ISA‐51 [92].

Based on previous studies, the effect of Montanide on enhancing the neutralizing capacity of vaccines is evident. Conversely, since a Th2 immune response after immunization can result in ADE, combining this adjuvant with CpG ODN helps promote a Th1 immune response.

## 4 Polysaccharide Adjuvant Systems

### 4.1 Delta Inulin (Advax)

Delta inulin (DI) is more effective at higher temperatures, exhibiting optimal stability and immunostimulatory activity within a temperature range of approximately 40°C–45°C, which aligns well with physiological body conditions. Such thermostability enhances its suitability for vaccine formulations, especially in contexts where maintaining the cold chain is challenging. It activates components of the immune system and, when combined with antigens, elicits a potent adaptive immune response that includes both humoral and cellular immunity [93, 94, 95]. Experiments have shown that combining DI and Japanese encephalitis virus (JEV) with inactivated influenza vaccines enhances both Th1 and Th2 responses, thereby boosting the immune system [93, 96]. When using a combination of a new recombinant spike protein or an inactivated SARS‐CoV vaccine with DI combined with CpG oligonucleotide, the serum neutralizing antibody titer increased without causing lung damage, in contrast to the results of a similar study using alum [52].

## 5 Agonist Adjuvant Systems

### 5.1 Toll‐Like Receptors

Pattern recognition receptors (PRRs) and toll‐like receptors (TLRs) detect pathogen‐associated molecular patterns (PAMPs) present at the cellular and endosomal levels. Binding with the appropriate ligand triggers the release of pro‐inflammatory cytokines and type 1 IFNs and activates innate immune cells, facilitating the induction of humoral and cellular responses [97].

TLR ligands have been used in many human and veterinary vaccine studies for infectious diseases [98, 99, 100]. Due to the different signaling pathways of these ligands, various immune responses are elicited (Th1/Th2/Th0). The TLR ligands studied include TLR3 (dsRNA), TLR4 (LPS), TLR5 (Flagellin), TLR7 (ssRNA), TLR8 (ssRNA), and TLR9 (unmethylated CpG oligonucleotide) [98]. Only the TLR4 ligand has been approved for use in human vaccines, such as those for hepatitis and malaria. However, additional TLR ligands are likely to be used in the future and may be incorporated into human vaccine formulations. Since alum has been shown to induce eosinophil infiltration into the lungs of vaccinated animals, more focus has been placed on TLR ligand experiments with SARS‐CoV inactivated virus vaccines. Experiments have shown that TLR ligands in the vaccine have remarkably protected animals from eosinophil infiltration into the lungs [101].

Intranasal administration of TLR3 agonists induces the production of IFN‐β and IFN‐γ, thereby protecting animals against SARS‐CoV infection. Therefore, the use of TLR3 agonists in older or immunocompromised individuals should be further investigated [102]. Intranasal vaccination offers benefits, such as reducing the risk of systemic inactivation of the vaccine and preventing lung damage. Following administration of the SARS‐CoV and CpG inactivated virus vaccine, local IgA secretion and neutralizing antibodies were observed in the serum. This response may enhance safety [98]. Other experiments have shown an increase in IgG antibody levels in serum and elevated IgA antibody levels in both serum and mucosal secretions after intranasal administration of the SARS‐CoV inactivated virus vaccine with CpG ODN 2006 [103]. The polyinosinic‐polycytidylic acid (PIKA) ligand has shown similar results when used with the SARS‐CoV inactivated virus vaccine, inducing high levels of both mucosal and serum antibodies [104].

On the other hand, in experiments with chimeric VLP vaccines on mice, the effectiveness of this agent was greater than the alum adjuvant. In these experiments, it prevented the pathogen from infecting susceptible cells [105]. When CpG ODN and R848 ligands were used with HLA‐A*0201‐restricted SARS‐CoV S epitopes, all three agonists increased the population of epitope‐specific CD8+ T cells, with the most pronounced effect observed for CpG ODN. The induced immune response was reported to exhibit a memory cell phenotype with long‐term persistence [106]. Spike protein‐based vaccines that use alum adjuvants have shown signs of lung damage. The use of TLR ligands that promote a Th1 response can reduce the risk of ADE. Additionally, a formulation combining a novel S protein or an N‐terminal domain of MERS‐CoV with alum and CpG induced high IgG2a antibody titers and IFN‐γ production [107, 108].

## 6 Recombinant Adjuvant Systems

### 6.1 Recombinant Onchocerca Volvulus Activation Associated Protein‐1 (rOv‐ASP‐1)

Experiments have shown that many helminth molecules, such as ASP‐1 found in the worm Onchocerca volvulus, have potent immunostimulatory effects. A combination of ASP‐1 and vaccine antigens has been shown to elicit both Th1 and Th2 responses. This response can be dominated by either type depending on the antigen [109, 110]. Thus, this safe adjuvant can be incorporated into vaccine formulations. It should be noted that the induction of responses to this factor has not affected its subsequent effectiveness [111]. In vaccines such as influenza, the use of ASP‐1 has increased IgG1 and IgG2a antibody levels. Its positive effect has been even more significant than that of adjuvants such as alum and trehalose dicorynomycolate (TDCM) [109, 112, 113]. A new combination of ASP‐1 protein and a novel SARS‐CoV spike protein antigen or an RBD‐based vaccine has elicited a complex immune response, predominantly Th1 [109]. Similar efficacy has been observed in the combination of rOv‐ASP‐1 and SARS‐CoV S protein or HIV‐1 gp120 [114]. Also, immunization with RBD SARS‐CoV plus rOv‐ASP‐1 in NHPs caused the generation of high titers of anti‐RBD neutralizing antibodies and low levels of anti‐rOv‐ASP‐1 antibodies [111].

## 7 Nano‐Adjuvant Systems

Nanomaterials are substances with particles ranging from 1 to 100 nm [115]. Nanoparticles (NPs) comprise a wide range of substances with specific physicochemical properties that can have immunostimulatory effects. However, a precise understanding of how the physicochemical properties of NPs influence the immune system remains unclear [116]. Today, biomedicine widely uses NPs for drug and gene delivery, vaccines, imaging, and medical devices, among other applications [117, 118]. Their ability to be engineered is one of their key features. Studies have shown that NP engineering can control the physicochemical properties of nanomaterials. In addition to causing increased reactive oxygen species (ROS), NP engineering also regulates the size and modulates immune responses to antigens [119, 120]. The higher the antibody titer, the better the vaccine's therapeutic effects. Of course, adverse effects associated with high‐titer vaccines must also be considered [121]. Researchers have reported that new vaccines based on NPs have stimulating effects on the immune system. As a result, it is anticipated that nano‐vaccines will function as a viable substitute for conventional vaccines [122]. NP‐derived vaccine adjuvants have many advantages over older models. These advantages include slow release, improved vaccine efficacy, and strong induction of humoral and cellular responses [123]. In recent years, immunology researchers have frequently added nanomaterials, known as nanoadjuvants, to vaccines. Typically, scientists classify and use NPs as vaccine adjuvants in two ways: as inorganic NPs, such as silver or gold, or as organic NPs, such as polymer particles. Researchers have paid special attention to NPs as adjuvants because they can both stabilize vaccine antigens and act as adjuvants [124]. Animal studies have shown that spike protein NPs, gold (Au) NPs, and hollow polymer NPs have significant potential to enhance the immune system against the coronavirus [125]. In 2020, a study used AuNPs as an adjuvant for UV‐inactivated SARS‐CoV vaccines. The combination of protein S adjuvant and AuNPs can elicit a robust antigen‐specific IgG response against SARS‐CoV infection; however, they do not produce protective antibodies [126]. Various nanomaterials, including aluminum salts, graphene, silica NPs, AuNPs, liposomes, and polymerized NPs, have been reported as vaccine adjuvants [127, 128].

## 8 Other Adjuvant Systems

### 8.1 Protollin

Protollin is a preparation of LPS proteosomes derived from Shigella flexneri. It should be noted that the proteosomes themselves contain hydrophobic outer membrane proteins derived from Neisseria meningitidis. TLR2 and TLR4 signaling are involved in the function of Protollin, resulting in the production of cytokines and IFNs, as well as activation of APCs. Intranasal administration of Protollin with influenza antigen in mice elicited serum IgG and mucosal secretory IgA and conferred protection against the virus [129, 130]. It has also been shown to reduce allergic asthma and prevent pneumonia resulting from Shigella infection in mice [131, 132].

Moreover, this adjuvant plays an influential role in vaccines for healthy adults [133]. This adjuvant induces serum IgG and mucosal IgA in trivalent influenza vaccines [134]. In experiments on mice, a compound of this agent with a new combination of SARS‐CoV S protein infused high levels of serum IgG‐specific antigen and IgA antibodies, which caused a higher infusion than in similar conditions using intramuscular alum. Thus, it has shown effectiveness against the virus in this study [135]. As the experiments show, the use of the Protollin adjuvant in the development of the optimal SARS‐CoV‐2 vaccine should be considered more and more.

## 9 Emerging Vaccine Platforms and Novel Adjuvant Strategies

Rapid development of SARS‐CoV‐2 vaccines has introduced novel platforms, notably mRNA vaccines. Pfizer's BNT162b2 and Moderna's mRNA‐1273 use lipid nanoparticle (LNP) systems that not only protect the mRNA but also activate innate immune responses by enhancing cytokine production and antigen presentation [136, 137]. Moreover, adenoviral vector‐based vaccines such as AstraZeneca's ChAdOx1 and Johnson & Johnson's Ad26.COV2.S reflect additional trends in COVID‐19 vaccine innovation [138, 139]. Similarly, the NVX‐CoV2373 vaccine employs the Matrix‐M adjuvant to elicit a balanced immune response [140]. These advancements suggest that self‐adjuvanting delivery systems, merging traditional and nano‐adjuvant strategies, may offer strategic advantages for rapid, durable immunity [141].

## 10 Conclusions

The development of effective vaccines against SARS‐CoV‐2 remains essential for controlling the COVID‐19 pandemic. This review underscores the critical role of immunostimulatory adjuvants and nano‐adjuvants in enhancing vaccine efficacy by fostering robust humoral and cellular immune responses while enabling antigen dose sparing. Drawing upon insights from vaccines targeting related coronaviruses and encompassing a diverse array of adjuvant systems, including aluminum salts, oil‐in‐water and water‐in‐oil emulsions, polysaccharides, TLR agonists, recombinant proteins, and nanomaterials, this study highlights promising strategies to optimize immune activation and safety profiles. Although the precise contributions of humoral and cellular immune responses to sustained protection against SARS‐CoV‐2 require further clarification, evidence from analogous coronaviruses emphasizes the significance of balanced Th1‐biased immunity in mitigating immunopathology and enhancing durability.

Several adjuvants, such as CoVaccine HT, Matrix‐M™, and rOv‐ASP‐1, have demonstrated favorable safety and potent immunogenicity in both preclinical and clinical studies. Additionally, licensed adjuvants like MF59® and AS03 may expedite vaccine development and deployment owing to their well‐established immunological profiles. The potential of emerging adjuvants, including Protollin, to elicit both systemic and mucosal immunity against respiratory viruses merits thorough investigation within SARS‐CoV‐2 vaccine formulations. Earlier concerns regarding pathogenic responses associated with inactivated SARS‐CoV and MERS‐CoV vaccines have not been observed with SARS‐CoV‐2, particularly when adjuvants that promote Th1 responses, such as CpG 1018, are included.

In conclusion, the prudent selection and design of adjuvants capable of inducing balanced and durable immune protection with minimal adverse effects constitute a crucial avenue toward the development of optimal SARS‐CoV‐2 vaccines. Ongoing research that integrates conventional and nanotechnology‐derived adjuvant platforms will be pivotal in realizing safe, effective, and broadly protective vaccines to address current and future global health challenges.

## Author Contributions

**Peyman Kheirandish Zarandi:** conceptualization, writing – original draft preparation, data curation. **Mohammad Reza Zinatizadeh:** writing – original draft preparation, data curation, writing – reviewing and editing. **Mohsen Ghiasi:** data curation and figure. **Mohammad Rezaei Zadeh Rukerd:** writing – review and editing. **Hanieh Mirkamali:** writing – review and editing. **Ehsan Shokri:** project administration, supervision, conceptualization, writing – reviewing and editing. All authors contributed to the article and approved the submitted version.

## Disclosure

All authors have read and approved the final version of the manuscript. Ehsan Shokri had full access to all of the data in this study and takes complete responsibility for the integrity of the data and the accuracy of the data analysis.

## Conflicts of Interest

The authors declare no conflicts of interest.

## Transparency Statement

The lead author, Ehsan Shokri, affirms that this manuscript is an honest, accurate, and transparent account of the study being reported; that no important aspects of the study have been omitted; and that any discrepancies from the study as planned (and, if relevant, registered) have been explained.

## Data Availability

Data sharing is not applicable to this article as no new data were created or analyzed in this study.
